# Using deep learning models as a genetic architecture for the simulation of breeding schemes

**DOI:** 10.1093/g3journal/jkag187

**Published:** 2026-07-09

**Authors:** Olumide Onabanjo, Theo Meuwissen, Hans Magnus Gjøen, Emre Karaman, Thinh T Chu, Peer Berg

**Affiliations:** Department of Animal and Aquacultural Sciences, Norwegian University of Life Sciences, Ås 1432, Norway; Department of Animal and Aquacultural Sciences, Norwegian University of Life Sciences, Ås 1432, Norway; Department of Animal and Aquacultural Sciences, Norwegian University of Life Sciences, Ås 1432, Norway; Center for Quantitative Genetics and Genomics, Aarhus University, Aarhus 8000, Denmark; Center for Quantitative Genetics and Genomics, Aarhus University, Aarhus 8000, Denmark; Department of Animal and Aquacultural Sciences, Norwegian University of Life Sciences, Ås 1432, Norway

**Keywords:** genetic architecture, breeding schemes, simulation, deep learning

## Abstract

In several simulation studies, long-term selection led to the rapid depletion of genetic variance. These outcomes differ from real-life observations that we aim to replicate, thereby highlighting a fundamental limitation of current classical quantitative genetic simulation models. Deep learning (DL) models have demonstrated promising results in capturing complex interactions essential for maintaining genetic variance; thus, we hypothesize that DL-based genetic simulation models may preserve more genetic variance than classical models, because the biological pathways underlying complex traits exhibit interactions that classical models ignore. The primary objective of this study was to introduce alternative DL-based genetic simulation models and compare them with classical genetic simulation models in terms of their retention of additive genetic variance under truncation selection in a simulated full-sib pig breeding scheme using real haplotypes as founders. After 20 generations of directional truncation selection, the classical models (A, ADAA, and ADAAADDD) retained between 55% and 64% of their initial additive genetic variance. In contrast, while the DL_simple model lost all its additive variance, the DL medium retained 92% to 98% of its additive variance, and the DL_complex model's initial additive variance increased by 296% to 314%. This paper introduces DL-based genetic simulation models and concludes that their ability to retain additive genetic variance depends on the models' architectural complexity. When sufficiently complex, DL-based models exhibit greater retention of additive genetic variance because they intrinsically capture epistatic interactions that are converted into additive variance, as selection progresses, thus, affirming the role of non-additive genetic effects in maintaining long-term genetic variation.

## Introduction

Stochastic simulation softwares (Pook et al. 2020; Gaynor et al. 2021; Chu and Jensen 2025) provide a time-efficient and cost-effective approach to studying the long-term effects of directional selection in breeding schemes, enabling breeders to predict outcomes, optimize strategies, and avoid common pitfalls in real-world breeding programs (Hill and Caballero 1992). However, in several simulation studies, the long-term consequences of directional selection have been observed to lead to the rapid exhaustion of additive genetic variance. For example, genetic variance was exhausted in a simulated plant breeding program for winter wheat selected for 20 years (Gaynor et al. 2017), peas selected for 30 years (Atanda and Bandillo 2024) and North American barley selected for 50 years (Vanavermaete et al. 2020). In a simulated animal breeding scheme, Duenk et al. (2020) reported near exhaustion of variance, whereas Wientjes et al. (2022) reported a variance loss exceeding 75% after 50 generations of selection.

The rate of additive genetic variance depletion is high in classical genetic simulation models, and the results differ from observations in real-world selection experiments. For example, the Illinois maize experiment (Dudley and Lambert 2004) has been selected for increased oil and protein content, and yet genetic variation persists after 100 generations. Moreover, the selection for increased milk production in dairy cows (Miglior et al. 2017) and the commercial selection for growth rate in chickens (Havenstein et al. 1994) both began in the 20th century, and despite decades of selection, genetic variation persists in these populations to date (Sosa-Madrid et al. 2023). The persistence of genetic variation in real biological populations, despite intensive long-term directional selection, suggests a fundamental limitation of classical quantitative genetic simulation models, even when they allow for new mutations (Havenstein et al. 1994; Strucken et al. 2015; Hill 2016; Miglior et al. 2017; Zou et al. 2020; Wientjes et al. 2022).

Classical genetic simulation models have often overlooked non-additive genetic effects, particularly higher-order epistatic interactions, because explicitly modeling them is computationally complex. These epistatic interactions are ubiquitous in biological systems and play crucial roles in maintaining genetic variance; their absence in genetic simulation models might explain the observed disparities in the retention of genetic variance between real-world and simulated breeding schemes. Additionally, changes in allele frequencies due to selection can convert non-additive genetic variance into additive variance (Hallander and Waldmann 2007; Hill 2017), distributing selection pressure across multiple loci, thereby preventing rapid fixation. This variance conversion mechanism can continuously replenish additive genetic variance as selection proceeds (Neiman and Linksvayer 2006; Hallander and Waldmann 2007; Barton 2017; Hill 2017). Moreover, the polygenic architecture of complex traits involves thousands of variants with pleiotropic effects operating within interconnected biological networks. This network structure creates high-order epistatic interactions that simple additive models cannot capture (Uma et al. 2025).

As shown in recent studies (Onabanjo et al. 2025; Perelygin et al. 2025; van Hilten et al. 2025), Deep learning (DL) models offer a promising approach to capture biological complexities because they: (i) can learn complex nonlinear functions without requiring explicit specification of interaction terms and (ii) consist of hierarchical layers of neurons corresponding to biological processes with hierarchical layers, thus may show similar patterns of variance reduction under selection. Therefore, we hypothesize that DL models may better reflect the true underlying genetic architecture and, more specifically, that such complex genetic architectures retain genetic variance across multiple generations of selection in simulation studies by implicitly capturing epistatic interactions, nonlinear relationships, and network effects that characterize biological systems but are inadequately represented in classical quantitative genetic models, with their additive, dominance, and epistatic interaction effects.

Hence, the primary objective of this study was to introduce DL-based models as a complex genotype-to-phenotype map, i.e. as a genetic architecture, in simulations, and to compare these architectures with classical quantitative genetic models with respect to the retention of additive genetic variance across generations of selection. Additionally, we evaluated the rates of genetic response and inbreeding in the simulated populations.

## Materials and methods

### Founder population

The founder population used in this study was described in detail in Onabanjo et al. (2025). Briefly, the genotype and phenotype dataset of dam-line boars from Topigs Norsvin (van Son and Onabanjo 2025) served as the basis for simulations in this study and provided a founder population with realistic linkage disequilibrium and an allele frequency spectrum. The dataset comprises 13,944 boars from different generations, born between 2011 and 2023, within the Topigs Norsvin nucleus herd. The z-score normalized feed efficiency (FE) phenotype provided for all boars was already pre-corrected for environmental effects, including month of birth, herd-year birth, parity of dam, as well as random effects of pen and litter. Beagle (Browning et al. 2018) was used to perform genotype imputation and haplotype phasing. Genotype imputation achieved 50,000 SNP genotypes for all animals, and quality control was performed using PLINK (Purcell et al. 2007). The founders' genome consisted of 18 autosomal chromosomes of varying lengths and 35,977 biallelic SNPs distributed across them. A total of 12,000 SNPs were defined as QTLs, and effects following a normal distribution were simulated. The remaining SNPs were defined as markers, as shown in Table 1.

**Table 1.** Number of markers and QTLs per chromosome.

Chromosome number	Chromosome length (cM)	Number of SNP markers	Number of QTLs	Template loci
1	274.3	2637	1062	549
2	151.4	1566	771	303
3	132.7	1462	718	265
4	130.8	1590	757	261
5	104.5	1121	602	209
6	170.8	1903	940	341
7	121.8	1337	833	243
8	138.9	1419	796	277
9	139.5	1725	738	279
10	69.3	660	485	139
11	79.0	906	476	159
12	61.0	568	461	123
13	208.2	1637	683	417
14	141.7	1659	714	283
15	140.3	1613	694	281
16	79.4	933	515	159
17	62.1	629	365	125
18	55.8	612	390	111
Total	2161.5	23,977	12,000	4,524

Template loci are neutral, with adjacent loci separated by even, constant distances. Since they are not affected by the selection process, the IBD of these loci was used to calculate true inbreeding.

### Base and offspring populations

**Fig. 1.**
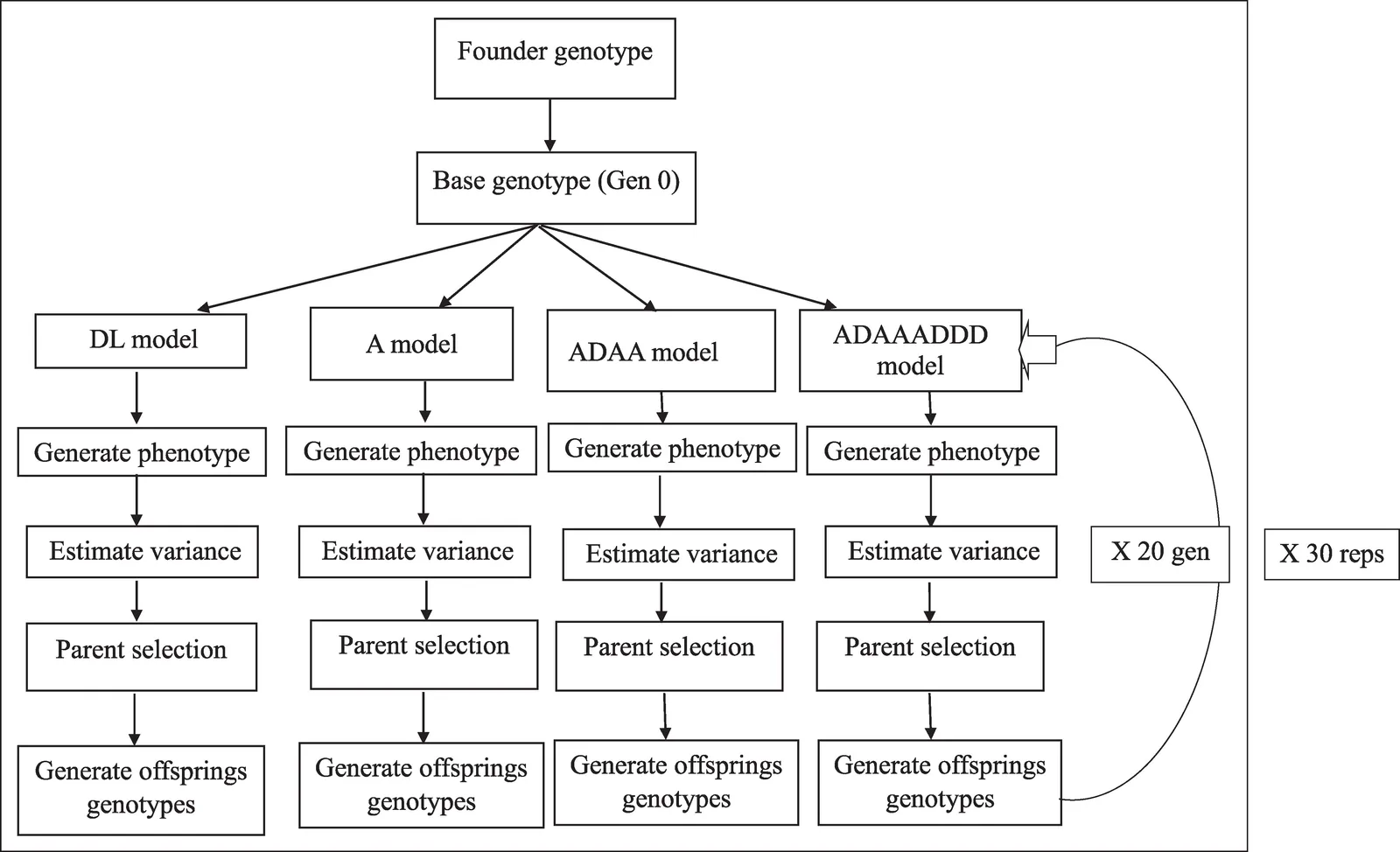
Schematic representation of the full-sib pig breeding scheme simulation.

From the phased founder population haplotypes, 800 individuals representing different generations were randomly selected. Of the randomly selected haplotypes, 200 were coded as male and 600 as female. ADAM-Multi (Chu and Jensen 2025) was used to sample the haplotypes, generating unique base individuals' genotypes. From these unique base individuals, 20 males and 200 females were randomly selected to serve as the base population (generation 0). Random mating was employed: each male mated with 10 females, and each female produced 10 offspring per mating, resulting in a total of 2,000 offspring per generation. Phenotypes were simulated for the offspring using different genetic architectures. Depending on the selection method employed, 20 males and 200 females were selected in each generation to be the parents of the next generation. This population was selected for 20 generations, and the process was replicated 30 times using different seeds. Figure 1 provides a schematic overview of the simulation process.

The genome of the simulated offspring reflects the structure and demographic history of the founders, comprising 18 autosomal chromosomes of varying lengths, bi-allelic QTLs, and markers. The inheritance patterns of markers and QTL alleles followed Mendelian inheritance, allowing recombination as in the ADAM software (Pedersen et al. 2009). A detailed explanation of the theoretical framework and simulation steps of ADAM-Multi can be found in the earlier versions of ADAM (Pedersen et al. 2009; Liu et al. 2019).

### Simulation of phenotypes using classical genetic models

A total of 12,000 SNPs were defined as QTLs and used to simulate phenotypes. These were the top SNPs that contributed to the additive variance of the feed efficiency trait, which was estimated using the DL model trained in our previous study (Onabanjo et al. 2025). The additive variance was calculated using $2\mathrm{pq}\beta^{2}$, where p and q are the frequencies of the alleles, respectively, and β is the average additive effect of each SNP across 50 independent splits of the training dataset. Effects β following a normal distribution were simulated for these QTLs.

Phenotypes were simulated using three classical genetic models with different assumptions and varying levels of complexity in genetic architecture. They include classical genetic simulation models with only additive effects (A), a model with additive, dominance, and additive-by-additive epistatic effects (ADAA), and a model with additive, dominance, and all pairwise second-order epistatic interactions (ADAAADDD). All simulation models were replicated 30 times. The models are mathematically expressed as


$$A\;\mathrm{model}:\;y=\mu +Z_{1}a+\varepsilon ,$$

$$\mathrm{ADAA}\;\mathrm{model}:\;y=\mu +Z_{1}a+\;Z_{2}d+Z_{3}e_{\mathrm{aa}}+\varepsilon ,$$

$$\mathrm{ADAAADDD}\;\mathrm{model}:\;y=\mu +Z_{1}a+\;Z_{2}d+Z_{3}e_{\mathrm{aa}}+Z_{4}e_{\mathrm{ad}}+\;Z_{5}e_{\mathrm{dd}}+\;\varepsilon ,$$

where **y** is a vector of phenotype, µ is the population mean, **a** is a vector of additive genetic effects, **d** is a vector of random dominance effects, $e_{\mathrm{aa}}$ is a vector of the second-order additive-by-additive epistatic effect, $e_{\mathrm{ad}}$ is a vector of the second-order additive-by-dominance epistatic effect, $e_{\mathrm{dd}}$ is a vector of the second-order dominance-by-dominance epistatic effect, ε is a vector of random residual errors sampled from a normal distribution and $Z_{1}$, to $Z_{5}$ are the corresponding design matrices with dimensions that assign observations to their vector of random effects. The random effects are assumed to follow a normal distribution with mean zero and variance,


$$\mathrm{Var}\;\left[\begin{array}{c} a\\ d\\ e_{\mathrm{aa}}\\ e_{\mathrm{ad}}\\ e_{\mathrm{dd}}\\ \varepsilon \end{array}\right]\;=\;\left[\begin{array}{cccccc} G\sigma_{a}^{2} & 0 & 0 & 0 & 0 & 0\\ 0 & D\sigma_{d}^{2} & 0 & 0 & 0 & 0\\ 0 & 0 & E_{\mathrm{aa}}\sigma_{e_{\mathrm{aa}}}^{2} & 0 & 0 & 0\\ 0 & 0 & 0 & E_{\mathrm{ad}}\sigma_{e_{\mathrm{ad}}}^{2} & 0 & 0\\ 0 & 0 & 0 & 0 & E_{\mathrm{dd}}\sigma_{e_{\mathrm{dd}}}^{2} & 0\\ 0 & 0 & 0 & 0 & 0 & I\sigma_{\varepsilon }^{2} \end{array}\right],$$

where $\sigma_{a}^{2}$ is the additive genetic variance, $\sigma_{d}^{2}$ is the dominance variance, $\sigma_{e_{\mathrm{aa}}}^{2}$ is the additive-by-additive epistatic variance, $\sigma_{e_{\mathrm{ad}}}^{2}$ is the additive-by-dominance epistatic variance, $\sigma_{e_{\mathrm{dd}}}^{2}$ is the dominance-by-dominance epistatic variance, $\sigma_{\varepsilon }^{2}$ is the residual variance, I is an identity matrix. G, D, $E_{\mathrm{aa}}$, $E_{\mathrm{ad}}$ and $E_{\mathrm{dd}}$ are the additive, dominance, additive-by-additive, additive-by-dominance, and dominance-by-dominance genomic relationship matrices that were constructed using the covariates for functional effects, as detailed in Chu et al. (2024) and Chu and Jensen (2025). Note that we set $\sigma_{\varepsilon }^{2}$ to a negligible number (0.01); therefore, our phenotypes equal approximately the total genetic value (TGV).

The phenotypic variance was calculated by summing up all variance components as: $\sigma_{p}^{2}\;=\;\sigma_{a}^{2}\;+\;\sigma_{d}^{2}\;+\;\sigma_{e_{aa}}^{2}\;+\;\sigma_{e_{ad}}^{2}\;+\;\sigma_{e_{dd}}^{2}\;+\;\sigma_{\varepsilon }^{2}$. The variance explained by additive variations relative to phenotypic variance is defined as narrow-sense heritability $(h^{2})\;=\;\frac{{\;\sigma }_{a}^{2}}{\sigma_{p}^{2}}$, dominance ratio $(d^{2})\;=\;\frac{{\;\sigma }_{d}^{2}}{\sigma_{p}^{2}}$, additive-by-additive epistasis ratio ${(e}_{\mathrm{aa}}^{2})\;=\;\frac{\sigma_{e_{\mathrm{aa}}}^{2}}{\sigma_{p}^{2}}$, additive-by-dominance epistatic ratio ${(e}_{\mathrm{ad}}^{2})\;=\;\frac{\sigma_{e_{\mathrm{ad}}}^{2}}{\sigma_{p}^{2}}$, and dominance-by-dominance epistatic ratio ${(e}_{\mathrm{dd}}^{2})\;=\;\frac{\sigma_{e_{\mathrm{dd}}}^{2}}{\sigma_{p}^{2}}$. Table 2 presents the input parameters for the variance components across different simulation scenarios for the classical genetic simulation models. Note that all scenarios have different narrow-sense heritabilities due to varying complexity/phenotypic variance, but they all have a broad-sense heritability of 1. A broad-sense heritability of 1 was achieved for all scenarios because $\sigma_{\varepsilon }^{2}$ is a negligible number; thus, the phenotypic variance ($\sigma_{p}^{2}$) for each scenario is comparable to the total genetic variance ($\sigma_{G}^{2}$).

**Table 2.** Values of variance components and heritabilities for the simulated phenotypes.

Model	$\sigma_{a}^{2}$	$\sigma_{d}^{2}$	$\sigma_{e_{\mathrm{aa}}}^{2}$	$\sigma_{e_{\mathrm{ad}}}^{2}$	$\sigma_{e_{\mathrm{dd}}}^{2}$	$\sigma_{G}^{2}$	$h^{2}$
**A**	0.356	-	-	-	-	0.356	1
**ADAA**	0.356	0.070	0.070	-	-	0.496	0.717
**ADAAADDD**	0.356	0.070	0.070	0.070	0.070	0.636	0.560

Since the residual variance $\sigma_{\varepsilon }^{2}$ was set to 0.01; $\sigma_{G}^{2}$ equals to $\sigma_{P}^{2}$, and $H^{2}\;=\;1$ for all models. $\sigma_{G}^{2}$ is the total genetic variance and $\sigma_{P}^{2}$ is the phenotypic variance. For comparisons between genetic models, it is essential to consider that genetic variance differed.

ADAM-Multi samples, centers, and rescales QTL effects to achieve these user-defined variances to generate their respective functional effects. The QTL effects were then kept constant across generations.

### Simulation of phenotypes using DL models

The training process of DL models is detailed in our previous study (Onabanjo et al. 2025). Our goal was to use the multilayer perceptron (MLP) model trained in our previous study (denoted as DL_simple) to simulate phenotypes; however, we increased the model's architectural complexity to enable it to learn the complex interaction landscape in the training dataset, resulting in the DL_medium and DL_complex models having a total of 224 and 896 neurons in their hidden layers, respectively. We simulated phenotypes using three trained DL models with different hyperparameters and varying levels of architectural complexity (DL_simple, DL_medium, and DL_complex), as detailed in Table 3. The DL model phenotype was simulated as


y=TGV+ε,

where TGV is a vector of total genetic values generated by the DL models and ε is a vector of negligible positive residual values ε∼N(0,0.01). Note that DL models are nonparametric; hence, we only control total genetic variance ($\sigma_{G}^{2}$) of the TGV via scaling and do not specify variance of different genetic components. All DL models were multilayer perceptron (MLP) networks trained on the founder population dataset provided by Topigs Norsvin. The DL models were trained using the 12,000 QTLs used to simulate phenotypes in classical models. The trained weights for DL-based models were saved and held constant across generations, thereby acting as a complex genotype-to-phenotype map.

**Table 3.** Hyperparameters for training DL models.

	DL_simple	DL_medium	DL_complex
Number of hidden layers	4	3	3
Number of neurons per hidden layer	96–64–32–16	128–64–32	512–256–128
L1 regularizer	0.007	0.0007	-
L2 regularizer	0.15	0.15	-
Hidden layers activation function	Leaky ReLu	Leaky ReLu	Leaky ReLu
Output layer activation function	Linear	Linear	Linear
Optimizer	Adam	Adam	Adam
Learning rate	0.00001	0.00001	0.00001
Loss function	MSE	MSE	MSE
Number of epochs	200	200	200
Early stopping	True	True	True
Batch size	32	32	32

All models were trained using multi-layer perceptrons (MLP) neural networks. The complexity of DL models is affected by both the increasing number of hidden layers and the increasing number of neurons per hidden layer. The increasing number of neurons across DL models increases the model's trainable parameter count, thereby enhancing its ability to learn complex interactions.

The phenotype simulation steps using DL models are, thus,

1. The founder dataset, supplied by Topigs Norsvin, was partitioned into training (75%) and validation (25%) subsets, and used to train the DL models using the 12,000 QTL genotypes as explanatory variables and the pre-corrected FE values as the response variable. Each model was trained once and ran 30 times in replicated simulations.
2. The trained weights of the QTLs for each replicate were saved and held constant to simulate TGV across generations, while the genotypes changed via standard Mendelian inheritance,
  TGV=f(X)*K,
  where f() denotes a trained model, X denotes the QTL genotype matrix of the simulated individuals, and K is a scaling factor to ensure that the total genetic variance ($\sigma_{G}^{2}$) of the TGV in the base population is 0.636, making it comparable to the ADAAADDD model, i.e. $K^{2}=\;\frac{0.636}{\mathrm{var}(f(X_{base})}$, where $X_{base}$ is the QTL genotype matrix from the base population animals.
3. Phenotypes were obtained by adding a normally distributed residual to the TGV,
  y=TGV+ε.
  Note that our residual value ε∼N(0,0.01) was small and negligible; therefore, the phenotype was comparable to TGV. For comparisons between genetic models, it is essential to consider that genetic variance differed.

### Estimation of variance components

REML estimation of variance components for all the generated data in the simulation models was performed using the DMUai module of the DMU package (Madsen et al. 2014). The univariate animal mixed model, including random additive-by-additive epistatic effect, was used to estimate variance components for all genetic models except the A model, which had only random additive genetic effects. In preliminary analysis, including dominance variance did not change the trends in additive or total genetic variance but frequently caused convergence failures and substantially increased computational time; we, therefore, excluded dominance effects from the variance component estimation models and focused on a model with additive and additive-by-additive components. The model with random additive-by-additive epistatic effect is expressed as


$$y=\mu +Za+We_{aa}+\varepsilon ,$$

where y is a vector of phenotype, μ is the population mean, a is the vector of random additive genetic effects $a\;∼\;N(0,\;G\sigma_{a}^{2})$, $e_{aa}$ is the vector of random additive-by-additive epistatic genetic effects $e_{aa}\;∼\;N(0,\;E\sigma_{e_{aa}}^{2})$, ε is a vector of residual values $\varepsilon \;∼\;N(0,\;I\sigma_{\varepsilon }^{2})$, **Z** and **W** matrices are design matrices that relate individuals to their respective additive genetic effects.

The genomic relationship matrix (**G**) was constructed from QTL genotypes using method 1 of VanRaden (2008). This was computed using the AGHmatrix package in R (Amadeu et al. 2023). The additive-by-additive epistatic matrix (**E**) was derived from the Hadamard product of the additive genomic relationship matrix, as described by Vitezica et al. (2017).

The variance components were estimated within each generation using phenotypic data from the previous three generations; thus, we used 6,000 individuals in each generation, except for the first two, which used 2,000 and 4,000 individuals, respectively, to approximate the variance structure around that time point while keeping computation feasible.

### Selection methods

This study used three selection methods in its simulated populations: (i) GEBV: genomic selection, which is expected to rapidly deplete the additive genetic variance, (ii) TGV: highly accurate genomic selection, which is expected to result in rapid depletion of total genetic variance; and (iii) RANDOM: random selection, where genetic variance is merely lost due to inbreeding and genetic drift. Truncation was applied to both the GEBV and TGV to select the next-generation parents (the top 20 males and the top 200 females). In the RANDOM selection method, 20 males and 200 females were randomly selected to serve as parents of the next generation. This selection method was only used as a baseline for comparison.

Genomic prediction of breeding values was carried out using the DMU4 module of the DMU package (Madsen et al. 2014). The univariate animal mixed model used to estimate GEBV is similar to that used for variance estimation, except that it excludes the epistatic effect component and uses SNP markers to construct the genomic relationship matrix. Note that GBLUP requires a prior narrow-sense heritability value to estimate GEBV. This prior is unknown and estimated, introducing differences between scenarios because they have varying narrow-sense heritability as shown in Table 2. Also, the GEBV selection model ignores non-additive genetic effects.

### Metrics for evaluating simulation models

Genetic simulations of breeding schemes are primarily conducted to compare alternative genetic architectures and breeding strategies with respect to genetic gains and the loss of genetic variance across traits, time horizons, and inbreeding rates. These metrics provide insight into the short-term effectiveness and the long-term sustainability of breeding strategies. In our simulation study, we evaluated all six simulation models for the following metrics:


$$\mathrm{Proportion}\;\mathrm{of}\;\mathrm{variance}\;\mathrm{reduction}\;=\;\frac{\sigma_{GV_{t_{1}}}^{2}-\;\;\sigma_{GV_{t_{20}}}^{2}}{\sigma_{GV_{t_{1}}}^{2}},$$

$$\mathrm{Rate}\;\mathrm{of}\;\mathrm{decreasing}\;\mathrm{variance}\;\mathrm{per}\;\mathrm{unit}\;\mathrm{genetic}\;\mathrm{gain}\;=\;\frac{\Delta \sigma_{GV}^{2}}{\Delta G},$$

$$\mathrm{Rate}\;\mathrm{of}\;\mathrm{genetic}\;\mathrm{gain}\;\mathrm{per}\;\mathrm{genetic}\;\mathrm{standard}\;\mathrm{deviation}\;\mathrm{unit}\;=\;\frac{\Delta G}{\sqrt{\sigma_{G_{t_{1}}}^{2}}},$$

$$\mathrm{Rate}\;\mathrm{of}\;\mathrm{genetic}\;\mathrm{gain}\;\mathrm{per}\;\mathrm{percentage}\;\mathrm{rate}\;\mathrm{of}\;\mathrm{inbreeding}\;=\;\frac{\Delta G}{\text{\%}\Delta F},$$

where $\sigma_{GV}^{2}$ is the observed genetic variance, $t_{1}$ is the first generation, $t_{20}$ is the twentieth generation, *G* is genetic gain, and %ΔF is the average rate of inbreeding per generation estimated using $100*(1-\;e^{slope})$. True inbreeding was calculated using the IBD of the template loci. These templates were neutral loci, with adjacent loci having even, constant distances. At a template locus, the true inbreeding is the probability that two alleles randomly picked from the locus are IBD (Mendoza and Haynes 1974). The true inbreeding coefficient of an individual was then the mean of the probabilities from all 4,524 template loci.

## Results

### Additive and non-additive genetic variance

**fig. 2.**
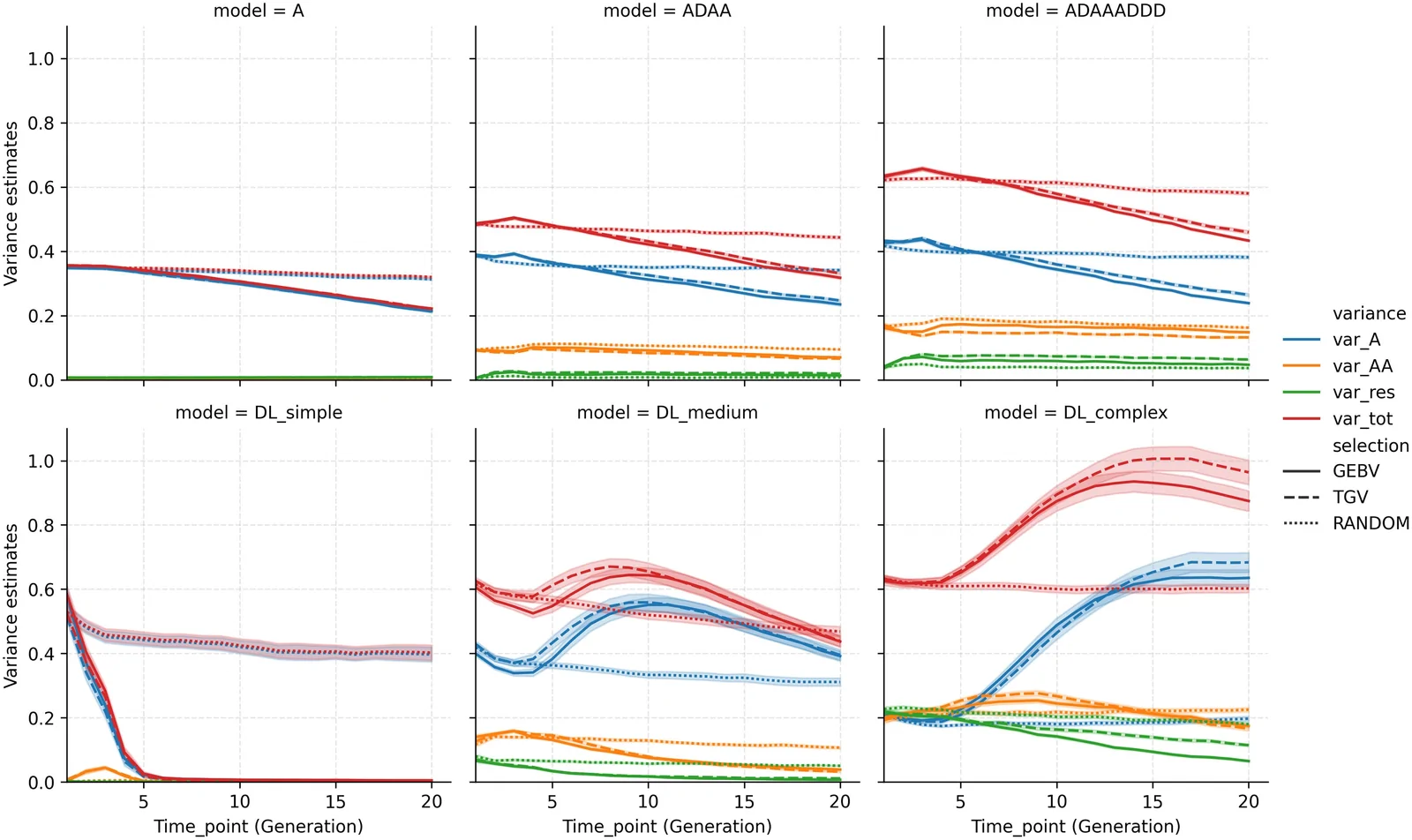
A line plot showing estimates of additive, additive-by-additive, residual, and total genetic variance over generations for six genetic simulation models under the GEBV, TGV, and RANDOM selection methods. The results shown are the averages and standard errors across 30 replicates, except for the DL_simple model, which had 10 replicates.

Figure 2 shows trends in REML-estimated additive ($\sigma_{a}^{2}$), additive-by-additive epistatic ($\sigma_{e_{\mathrm{aa}}}^{2}$), residual ($\sigma_{\varepsilon }^{2}$), and total genetic ($\sigma_{a}^{2}\;+\;\sigma_{e_{\mathrm{aa}}}^{2}+\;\sigma_{\varepsilon }^{2}$) variances for the six simulation models over 20 generations of GEBV, TGV, and RANDOM selection. Additionally, the summary of variance retained by each simulation model under the selection methods is shown in Table 4. Since our simulated phenotype was equal to the total genetic value, the estimated residual variance is the genetic variance not explained by the estimated additive and additive-by-additive epistatic effects; therefore, these values may reflect dominance or higher-order interactions that were not estimated.

**Table 4.** Summary of variance retained for six genetic simulation models under GEBV, TGV, and RANDOM selection.

		Retained variances (%)			
Selection method	Simulation model	Var_A	Var_AA	Var_res	Var_tot
GEBV	A	61.37	-	119.66	62.47
	ADAA	60.32	75.69	238.93	65.25
	ADAAADDD	55.10	91.69	113.83	68.18
	DL_simple	0.78	0.00	0.00	0.77
	DL_medium	97.93	27.69	9.94	72.10
	DL_complex	296.79	90.72	29.54	139.72
TGV	A	60.69	-	128.87	61.90
	ADAA	64.08	71.85	337.02	68.72
	ADAAADDD	62.10	77.61	172.89	72.66
	DL_simple	0.37	0.00	0.00	0.36
	DL_medium	92.01	25.31	15.92	70.03
	DL_complex	314.03	82.50	53.43	152.30
RANDOM	A	89.38	-	112.86	89.78
	ADAA	87.65	100.78	180.00	90.95
	ADAAADDD	91.52	98.33	91.15	93.31
	DL_simple	75.17	5100.13	34.84	75.99
	DL_medium	74.27	91.96	62.82	76.12
	DL_complex	94.06	114.75	79.31	95.18

For some DL scenarios, particularly DL_simple under RANDOM selection, the percentage of retained additive-by-additive variance exceeds 100% by a large margin (e.g. 5,100%). This arises because the initial AA variance is extremely small, so even small absolute changes translate into large percentage values; absolute AA variance remains relatively small.

Under GEBV and TGV selection, the A simulation model, which had only additive genetic effects, showed a linear decline in additive genetic variance, retaining 61.37% and 60.7% of its initial additive variance after 20 generations, respectively. The ADAA and ADAAADDD models, which included dominance and epistatic effects, retained 60.32% and 55.10% of their initial additive genetic variance under GEBV selection, and 64.1% and 62.1% under TGV selection, respectively. In contrast to classical genetic simulation models, DL-based models are nonparametric and partition the total initial variance into additive and non-additive components based on the model's architectural complexity. The DL_simple model, which partitioned almost all its initial total genetic variance as additive, lost all genetic variance by the fifth generation and maintained a near-zero genetic variance thereafter under both GEBV and TGV selection. The DL_medium model initially showed a slight decrease in additive genetic variance, followed by an increase that peaked at generation 10, then gradually declined. It retained 97.9% and 92.1% of its initial additive genetic variance under GEBV and TGV selection by generation 20. The epistatic and residual variances also reduced substantially under both selection methods. The DL_complex model, which had nearly the same initial additive and additive-by-additive genetic variances, showed increases of 296.1% and 314.0% in additive variance while retaining 90.7% and 82.5% of its initial additive-by-additive epistatic variance under GEBV and TGV selection, respectively.

Under RANDOM selection, all genetic simulation models retained considerably more variance than under directional selection. Additive variance retention ranged from 74.3% to 94.1% across all genetic simulation models, while total variance retention ranged from 76% to 95.2%, with classical models maintaining approximately 87.8% to 93.3% of their initial variance.

### Observed genetic variance

**Fig. 3.**
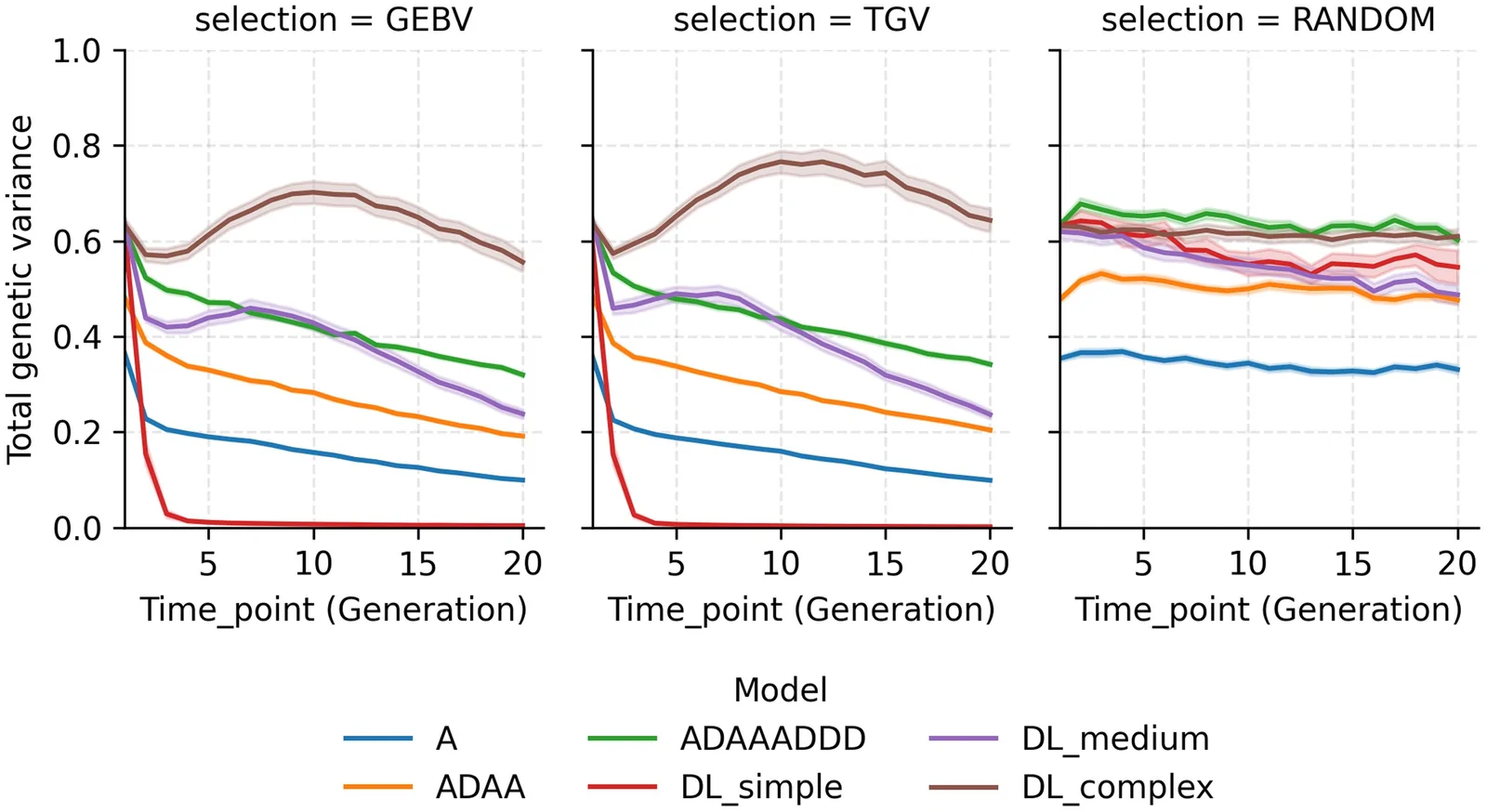
Observed total genetic variance for six genetic simulation models across generations of GEBV, TGV, and RANDOM selection. The results shown are the averages and standard errors of 30 replicates, except the DL_simple model, which had 10 replicates.

Figure 3 shows the observed total genetic variance across generations of GEBV, TGV, and RANDOM selection for all genetic simulation models. The genetic simulation models had different starting total genetic variances due to their varying levels of genetic architectural complexity. The A model had only additive genetic effects; thus, total genetic variance equals the additive genetic variance (0.356). The ADAA model accumulates the variances of additive, dominance, and additive-by-additive epistatic interactions, resulting in a total genetic variance of 0.496. The ADAAADDD model, which includes all second-order epistatic variances, yields a total genetic variance of 0.636. The total genetic variance of the DL models was scaled to match that of the ADAAADDD model for comparison.

Under GEBV and TGV selection, all classical genetic models showed a rapid decline in the observed total genetic variance. The A model declined sharply under both selection methods, retaining around 27% of their initial variance by generation 20. The ADAA model retained more variance than the A model, but still declined steadily, with retention at 39% and 42% under GEBV and TGV selection, respectively. The ADAAADDD model showed the highest variance retention among classical models, retaining 51% and 54% genetic variance under GEBV and TGV selection methods, respectively. Among the DL models, DL_simple exhibited the most extreme response, with total variance collapsing to near zero within five generations, indicating almost complete depletion of genetic variance under GEBV and TGV selection. In contrast, DL_medium retained 37% of genetic variance under both selection methods, while the DL_complex model showed an increase in total genetic variance, corresponding to 101% retention under TGV selection and 87% under GEBV selection.

Under RANDOM selection, total genetic variance declined slowly across all models, reflecting primarily the decay in variance due to inbreeding. Classical models retained 94% to 100% of their initial variance, with only minor fluctuations across generations, while DL models also showed high variance retention under RANDOM selection, with DL_simple, DL_medium, and DL_complex retaining approximately 78% to 96% of initial variance by generation 20.

### Genetic response to selection

**Fig. 4.**
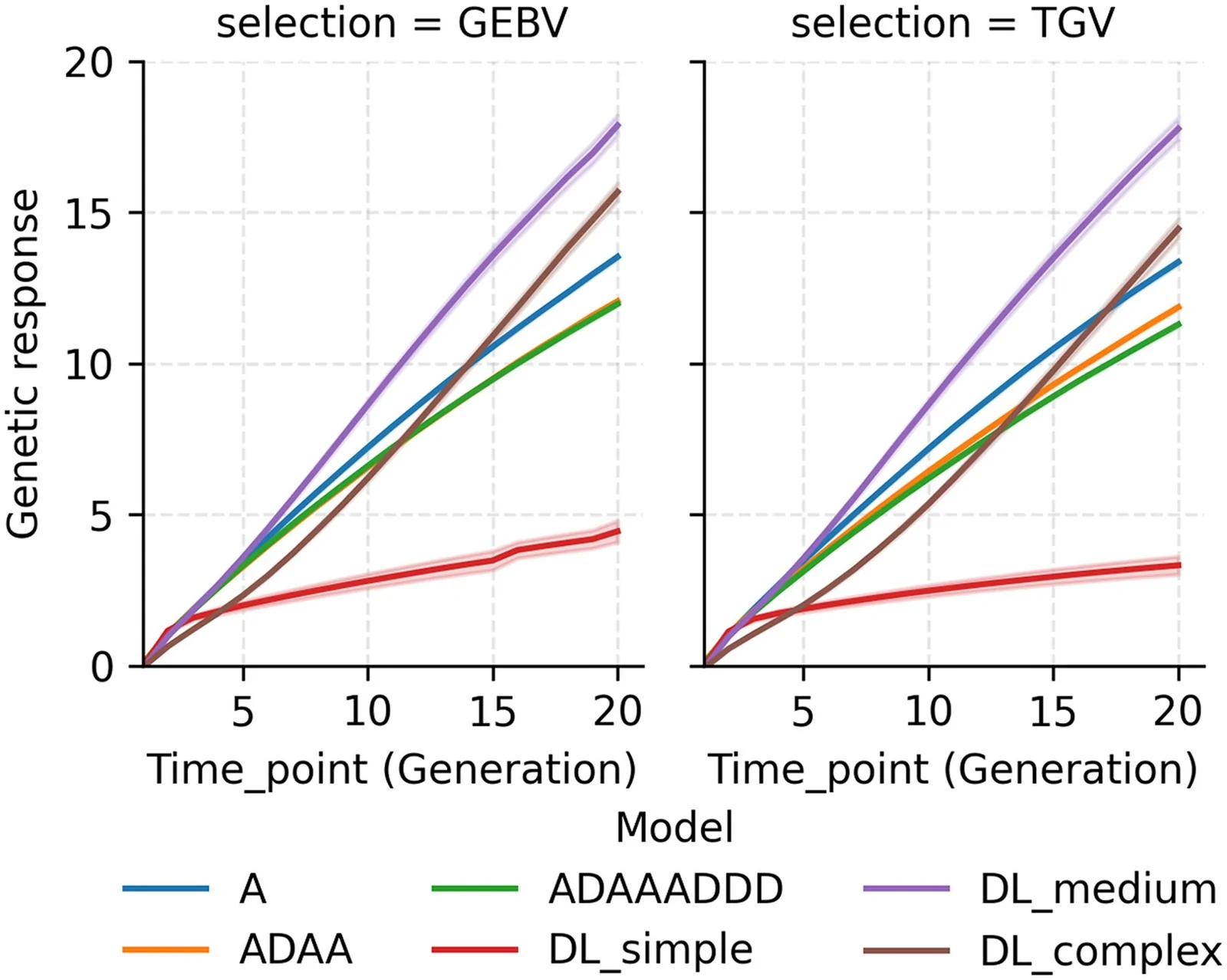
Cumulated genetic response for six genetic simulation models under GEBV and TGV selection. The plot for the RANDOM selection method was not shown as there was no response. The results shown are the averages and standard errors of 30 replicates, except the DL_simple model, which had 10 replicates.

Figure 4 shows the cumulative genetic response for six genetic simulation models across generations of GEBV and TGV selection. All simulation models showed a positive response to GEBV and TGV selection, but the magnitude varied substantially across models. The genetic responses of the classical genetic models to GEBV and TGV selection methods were moderate compared with those of the DL genetic simulation models. Of the classical simulation models under GEBV and TGV selection, the A model showed a higher response than the other classical models (ADAA and ADAAADDD), which had slightly lower gains. Additionally, including non-additive genetic effects in the classical genetic models did not increase genetic response in this simulation study; in fact, the genetic response of the classical models with non-additive effects was lower than that of the model with only additive effects, despite their higher initial total genetic variance. Among the DL models, DL_simple exhibited the lowest genetic response under the TGV selection method, with a slightly higher response under the GEBV selection. This lower response is consistent with the model's rapid loss of additive genetic variance. In contrast, DL_medium showed the largest response among all models under both selection methods. This was followed by DL_complex, which also responded strongly despite a slow start. Under the RANDOM selection method, genetic response remained near zero across all models (plot not shown), with no systematic increase over generations, indicating the absence of directional genetic change.

### Inbreeding

**Fig. 5.**
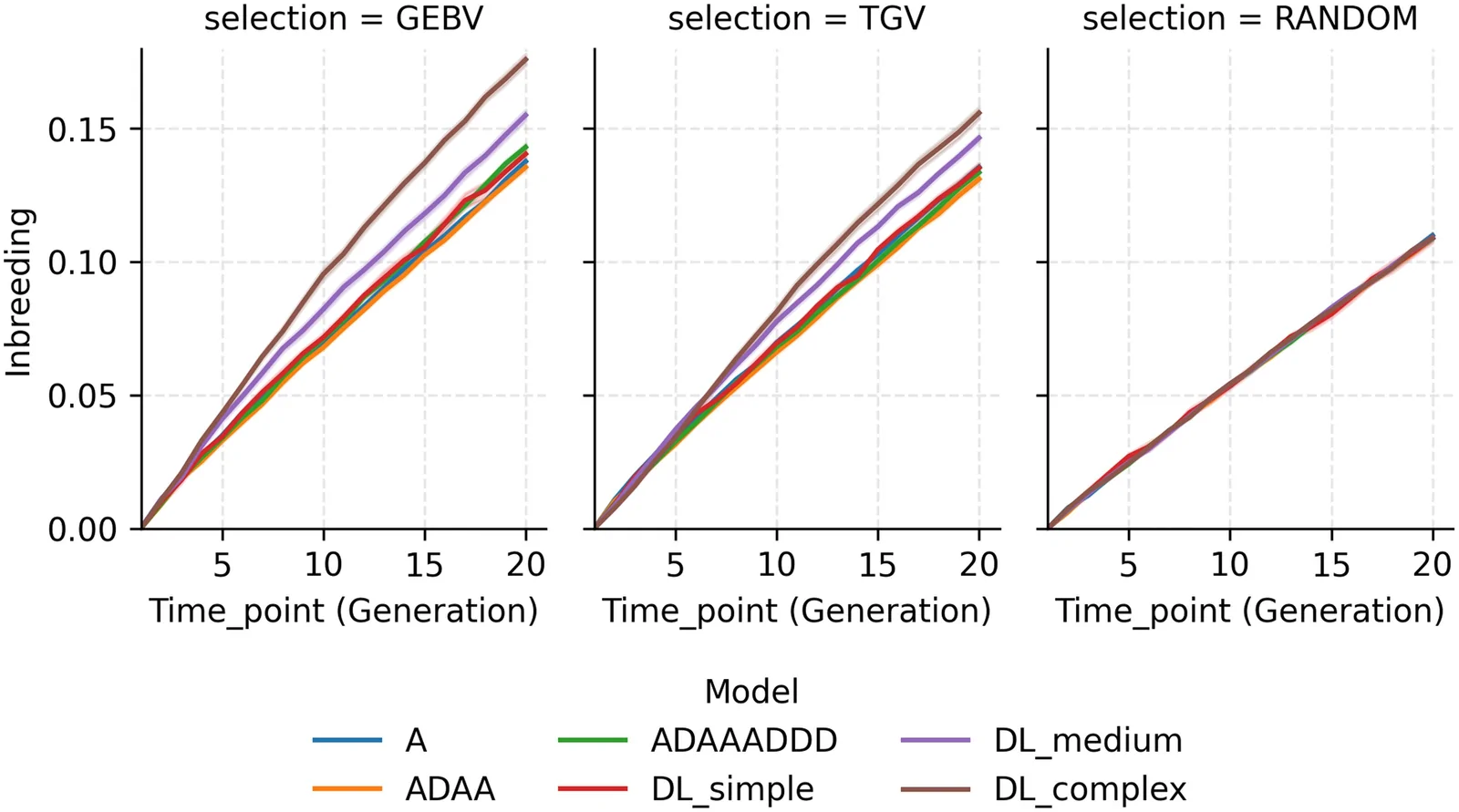
Average inbreeding for the six genetic simulation models under GEBV, TGV, and RANDOM selection. The results shown are the averages and standard errors of 30 replicates, except the DL_simple model, which had 10 replicates.

The average inbreeding over time, as shown in Fig. 5, illustrates the extent to which genetic diversity at neutral loci is lost due to finite population size and selection across generations. The inbreeding level increased over time across all genetic simulation models under GEBV, TGV, and RANDOM selection, but was consistently highest under GEBV selection. Under GEBV and TGV selection, the trend was similar: the DL models accumulated the highest inbreeding, with the DL_complex model showing the highest, followed by DL_medium and DL_simple, which had the lowest inbreeding among the DL genetic simulation models. The classical models all showed similar levels of inbreeding under GEBV and TGV selection, regardless of their genetic complexity. Under RANDOM selection, all simulation models reached the same level of inbreeding by generation 20, which is substantially lower than that under GEBV and TGV selection.

**Fig. 6.**
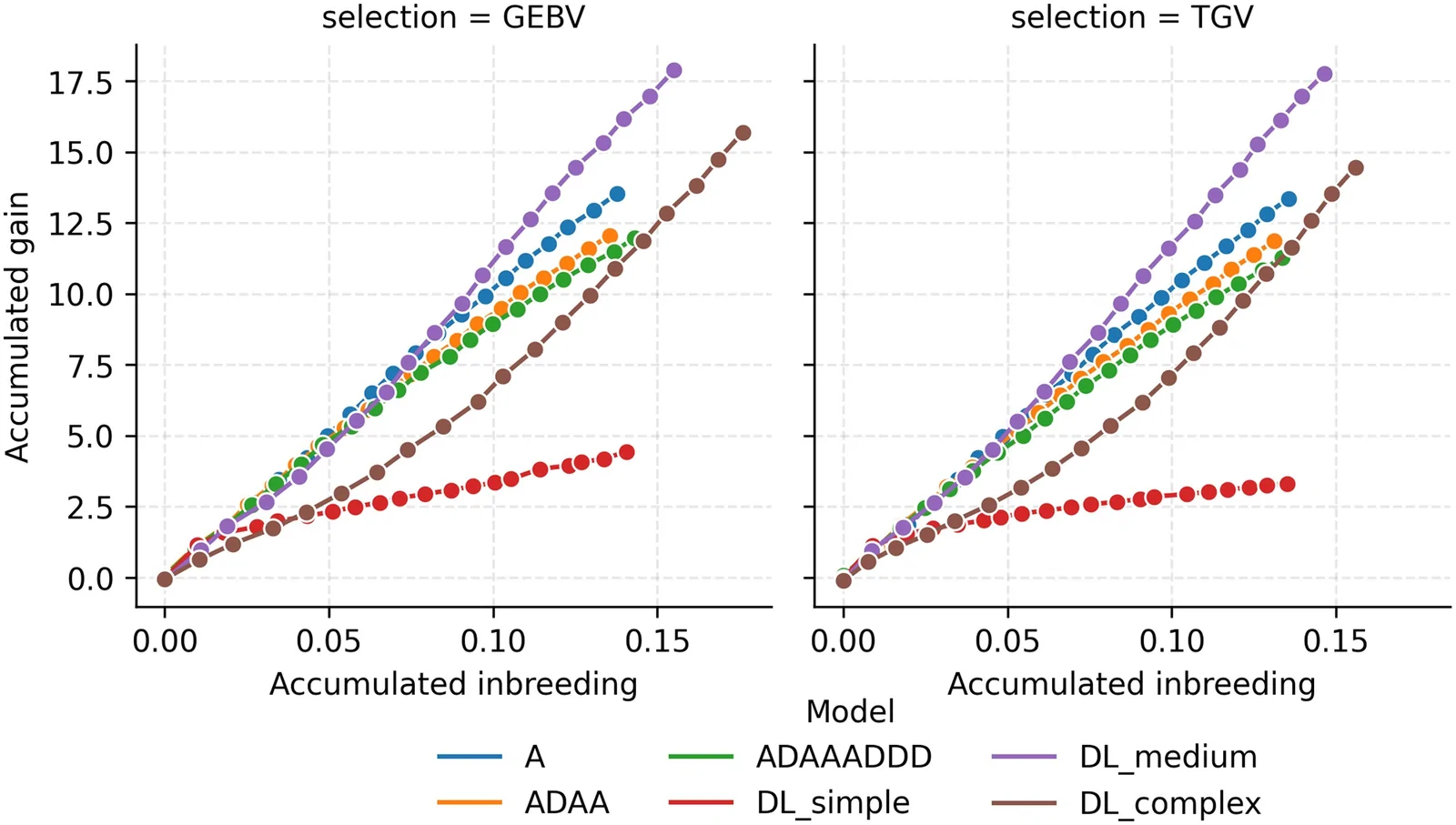
Trade-off curve of accumulated genetic gain vs accumulated inbreeding across selection time points for six genetic models under GEBV and TGV selection. The results shown are the averages and standard errors of 30 replicates, except the DL_simple model, which had 10 replicates.

The trade-off curve, also known as the combined efficiency plot, shown in Fig. 6, visualizes the balance between genetic gain and the loss of genetic diversity at neutral loci across various scenarios under GEBV and TGV selection. Under both selection methods, the DL_medium model yielded the highest genetic response per unit of inbreeding, while the DL_complex model also delivered a high genetic response, but at the expense of higher inbreeding levels. In contrast, the classical genetic models performed similarly under both selection methods, yielding moderate genetic responses for a given amount of inbreeding. Overall, the DL_simple had the lowest genetic response at inbreeding levels comparable to those of the classical models.

### Evaluation of models

The performances of the six genetic simulation models under GEBV and TGV selection methods were evaluated using four metrics, including the proportion of variance reduction, the rate of decreasing variance per genetic gain, the rate of genetic gain per genetic standard deviation unit, and the rate of genetic gain per percentage of inbreeding, as shown in Table 5.

**Table 5.** Evaluation of model performance based on key breeding metrics.

		Evaluation metrics			
Selection methods	Genetic simulation models	Proportion of variance reduction	Rate of decreasing variance per genetic gain	Rate of genetic gain per genetic standard deviation unit	Rate of genetic gain per percentage rate of inbreeding
**GEBV**	**A**	0.588 (0.008)	0.020 (0.001)	1.164 (0.010)	0.537 (0.007)
	**ADAA**	0.513 (0.009)	0.025 (0.001)	0.894 (0.008)	0.533 (0.006)
	**ADAAADDD**	0.396 (0.009)	0.027 (0.001)	0.775 (0.006)	0.520 (0.005)
	**DL_simple**	0.994 (0.001)	0.155 (0.013)	0.297 (0.021)	0.172 (0.013)
	**DL_medium**	0.601 (0.015)	0.023 (0.001)	1.197 (0.022)	0.633 (0.018)
	**DL_complex**	0.091 (0.025)	0.004 (0.001)	1.031 (0.014)	0.491 (0.012)
**TGV**	**A**	0.586 (0.009)	0.020 (0.001)	1.163 (0.012)	0.552 (0.007)
	**ADAA**	0.481 (0.011)	0.025 (0.001)	0.872 (0.006)	0.596 (0.006)
	**ADAAADDD**	0.364 (0.001)	0.027 (0.001)	0.726 (0.006)	0.592 (0.006)
	**DL_simple**	0.997 (0.001)	0.195 (0.014)	0.235 (0.020)	0.148 (0.013)
	**DL_medium**	0.604 (0.015)	0.023 (0.001)	1.180 (0.023)	0.703 (0.014)
	**DL_complex**	−0.022 (0.028)	−0.001 (0.001)	0.948 (0.018)	0.601 (0.017)

The results shown are the averages and standard errors of 30 replicates, except the DL_simple model with 10 replicates.

Across both selection methods, the classical genetic models showed moderate variance reduction and relatively stable efficiency values. Under GEBV selection, the additive model (A) showed a variance reduction of 0.588, which decreased progressively with increasing genetic complexity to 0.513 in ADAA and 0.396 in ADAAADDD. In contrast to the classical models, DL models had different trends. The DL_simple model lost almost all of its variance, consistent with its rapid collapse of additive variance; DL medium lost 0.601, whereas DL_complex exhibited no net loss of variance. Similar trends were observed under TGV selection, where variance reduction declined from 0.586 in A to 0.364 in ADAAADDD. These results indicate that inclusion of non-additive effects improved the retention of genetic variance during selection. The rate of decreasing variance per unit genetic gain was relatively low and stable across the classical models under both GEBV and TGV selection, ranging from 0.020 to 0.027, comparable to DL_medium (0.023). In contrast, DL_simple showed substantially higher rates under both GEBV (0.155) and TGV (0.195), whereas DL_complex showed the lowest rates, including a near-zero value under TGV selection (−0.001).

The rate of genetic gain per genetic standard deviation unit differed markedly among models. Under GEBV selection, the highest values were observed for DL_medium (1.197) and the additive model (1.164), whereas ADAA and ADAAADDD showed lower efficiencies (0.894 and 0.775, respectively). DL_simple had the lowest value (0.297). Similar patterns were observed under TGV selection, with DL_medium again achieving the highest efficiency (1.18) and DL_simple the lowest (0.235). DL_complex showed intermediate performance under both selection methods (1.031 under GEBV and 0.948 under TGV). The rate of genetic gain per percentage rate of inbreeding further highlighted differences in selection efficiency. Under GEBV selection, DL_medium showed the highest value (0.633), followed by the classical models (0.520 to 0.537). Under TGV selection, DL_medium again showed the highest efficiency (0.703), while ADAA and ADAAADDD exhibited intermediate values (0.596 and 0.592, respectively). DL_simple consistently showed the lowest efficiency across both selection methods (0.172 with GEBV and 0.148 with TGV).

Overall, the results reveal clear differences among model architectures. DL_simple performed poorly overall, marked by significant variance depletion, low genetic gain efficiency, and poor gain-to-inbreeding ratios. Classical models showed steady but moderate results, with greater genetic complexity enhancing variance retention but reducing gain efficiency. DL_medium consistently delivered the highest efficiency for genetic gain relative to both genetic variance and inbreeding, while DL_complex uniquely preserved genetic variance with minimal depletion, especially under TGV selection.

## Discussion

This study suggested using nonparametric DL-based models as a genotype-to-phenotype map, i.e. as a genetic architecture, and compared them with classical models in terms of retained additive genetic variance, genetic response, and accumulated inbreeding over 20 generations of selection. Our findings support our hypothesis that DL models may better reflect the true underlying genetic architecture and that such complex genetic architectures may retain more genetic variation than classical genetic models under directional selection. However, the DL model's ability to capture such interactions that maintain variation depends on its architectural complexity. A detailed discussion on the implications of the results, as well as limitations and future directions, is presented in the following sections. For comparisons between genetic models, it is essential to consider that genetic variance differed, as described in the materials and methods section. However, the DL models were scaled to match the ADAAADDD model's initial total genetic variance. Hence, comparisons of variance retention and response are most meaningful between these architectures.

### Variance retention of classical models

Directional selection resulted in a rapid, linear loss of additive genetic variance (Fig. 2), consistent with previous studies by Duenk et al. (2020), Esfandyari et al. (2017), Vanavermaete et al. (2020), and Wientjes et al. (2022). This additive variance loss was higher under the GEBV selection method than under the TGV selection method because this method exploits additive genetic effects, leading to faster fixation of favorable alleles and stronger depletion of additive variance. Under GEBV selection, we observed that the retained additive variance decreased with increasing complexity of classical models (Table 4). In the A model, the relatively high initial contribution of additive variance means that GEBV selection rapidly converts additive variance into genetic gain, resulting in moderate retention of additive variance (61.4%). In contrast, in the ADAA and ADAAADDD models, part of the genetic variance is distributed into epistatic and dominance interactions rather than remaining purely additive. As a result, although these models may start with substantial total genetic variance, the proportion attributable to additive effects is effectively lower. However, the non-additive interactions buffer the loss of total variance by maintaining variability in non-additive components, which is why ADAA and ADAAADDD retain less additive variance than A, despite retaining more total variance. On the other hand, we observed a higher loss of additive genetic variance in the A model and, to a lesser extent, in the ADAA and ADAAADDD models, under TGV selection.

There was no observed increase in additive variance due to non-additive variance in our classical models, consistent with the findings of Esfandyari et al. (2017). Several researchers have reported that the inclusion of epistatic interactions (Young 1967; Carlborg et al. 2006; Neiman and Linksvayer 2006; Hallander and Waldmann 2007; Barton 2017; Hill 2017; Wientjes et al. 2022) and dominance to a lesser extent (Fuerst et al. 1997) may release new additive variance, which buffers genetic variance in the long term. This was not the case in our study, possibly because the low ratio of additive-by-additive epistatic variance to additive variance limits the release of new additive genetic variance, which maintains variation in the long term. The initial ratio of our additive-by-additive epistatic variance to the additive variance was 1/5; however, Goodnight (1987) showed that the additive variance will not increase unless the ratio is at least 1/3.

### Variance retention of DL-based models

The DL models exhibited contrasting behaviors under directional selection, ranging from complete depletion of additive variance in DL_simple to substantial amplification in DL_complex. These models are nonparametric, and the partitioning of the initial total variance into additive and non-additive components is largely dependent on the model's architectural complexity. We observed that while the initial portion of non-additive variance learned by these models increased with architectural complexity, the initial portion of additive variance decreased (Fig. 2). These higher ratios of the initial non-additive variance, particularly the additive-by-additive epistatic interaction relative to the additive variance (∼1/3 in DL_medium and ∼1 in DL_complex), might explain the increased variance observed in the DL_medium and DL_complex models under directional truncation selection, as these ratios are in line with the minimum ratio reported by Goodnight (1987) to observe increased additive variance.

The variance increase observed is consistent with previous simulation studies by Hallander and Waldmann (2007) and Fuerst et al. (1997). They reported increased variance when using classical simulation models, with an initial additive-by-additive-to-additive variance ratio of at least 1/3. Although this new additive variance was only temporary, it eventually approached exhaustion after several generations of selection. This was not the case for the DL_complex model; therefore, we ran an additional simulation for 40 generations and observed a significant decline in additive genetic variance after generation 20 (see Supplementary Fig. 1). However, the model still retained 142% and 202% of its initial variance after 40 generations of GEBV and TGV selection, respectively.

Also, the increase in additive variance of the DL_complex model can be understood in terms of several interconnected biological mechanisms that the model may implicitly capture. First, the hidden layers in our DL genotype-to-phenotype map effectively act as a reservoir of dominance and epistatic variance. As selection shifts allele frequencies, the marginal effects of these hidden interactions change, thereby converting previously non-additive variance into accessible additive variance, consistent with the theoretical predictions of epistatic variance conversion described by Hill (2017). However, the magnitude of this increase (exceeding 100% of the initial total variance) suggests that the DL_complex architecture may model a system with decanalisation, in which the breakdown of robust networks under intense selection releases large amounts of cryptic variation (Tawfeeq et al. 2024). While beneficial for avoiding selection plateaus, this raises questions about the biological realism of the magnitude. Unlike the linear constraints of the ADAAADDD model, the non-linear activation functions in the DL model allow for the emergence of super-performers that drastically inflate phenotypic spread.

Second, the concept of genetic canalization, the ability of biological systems to produce consistent phenotypes despite genetic and environmental perturbations, provides a framework for understanding the maintenance of variance. Developmental systems exhibit nonlinear genotype–phenotype relationships where large (non-)genetic perturbations can be tolerated within certain ranges with minimal phenotypic effects, but changes beyond critical thresholds can have dramatic consequences. This nonlinearity creates phenotypic robustness while preserving underlying genetic variation (Green et al. 2017). For instance, genetic changes exceeding the critical thresholds will result in increased, rather than reduced, genetic variation. DL models, with their inherent capacity to model complex nonlinear functions, may naturally capture developmental stability mechanisms that maintain genetic variance by traversing alternative routes through the neural network, thereby depicting a stable genotype-to-phenotype map with buffering effects.

Third, real biological networks contain hierarchical gene regulatory structures with multiple layers of interaction that create distributed genetic effects (Hatleberg and Hinman 2021; Papadadonakis et al. 2024). Hub genes in genetic interaction networks often have tightly regulated expression that buffers against perturbations, while maintaining the capacity to release cryptic genetic variation under specific conditions (Park and Lehner 2013; Arcuschin et al. 2023). The layered architecture of neural networks may mirror these biological network hierarchies, in which intermediate layers capture different levels of genetic interaction and regulatory control, thereby preventing the rapid fixation of alleles that characterizes simpler additive models. Lastly, the phenomenon of epistatic variance conversion (Neiman and Linksvayer 2006; Hallander and Waldmann 2007; Barton 2017; Hill 2017), where non-additive genetic effects can be converted to additive variance during selection, provides a continuous source of genetic variation that maintains evolutionary potential. DL models, through their ability to implicitly capture higher-order epistatic interactions, model these variance conversion mechanisms that replenish genetic variation as selection proceeds (Cui et al. 2022).

On the other hand, the failure of the DL_simple model illustrates a critical trade-off in generative modeling. As shown in Table 3, this model had few neurons and utilised high L1 and L2 regularization (Lasso), which enforces sparsity. Consequently, the model likely learned a “quasi-additive” architecture that depends on a small subset of large-effect QTLs. Selection rapidly fixed these specific QTLs, and the variance collapsed (Table 4), mimicking the limitations of standard additive simulations with few loci.

### Response to selection

Our result showed that the DL_medium model, closely followed by the DL_complex model, exhibited the strongest response to directional truncation selection (Fig. 4), likely reflecting their ability to capture more complex epistatic interactions and nonlinear genotype-to-phenotype relationships than classical genetic models. In contrast, the DL_simple model showed the weakest response to directional truncation selection due to its rapid loss of genetic variance. Compared to the other models, the DL_complex model showed a slower response to selection in the first few generations, most likely due to its low initial additive variance. The accumulated response increased as the additive variance increases fuelled by the conversion of additive-by-additive epistatic variance into additive variance (Hill 2017). To confirm if this variance conversion will continually fuel genetic response, we ran an extra simulation for 40 generations. The result showed that the DL_complex model surpassed the DL_medium model in accumulated response by generation 40 (see Supplementary Fig. 2), further affirming the role of non-additive effects in long-term response to selection.

Among the classical models, the A genetic simulation model had the highest gain, consistent with Esfandyari et al. (2017), who compared the response to selection of four models (A, A/D, A/AA, and A/D/AA) over 100 generations of selection (phenotypic and BLUP-EBV), and reported that the A model had the highest response. The inclusion of non-additive genetic effects in the classical genetic models did not increase genetic response in our simulation study, consistent with previous research by Wientjes et al. (2022), Vitezica et al. (2018), and Momen et al. (2018). In contrast to our findings, Fuerst et al. (1997) reported that the A/D−/AA + model had the highest long-term selection response across all cases when they compared the response to phenotypic selection of eight models with three different initial heritabilities over 200 generations. Also, Hallander and Waldmann (2007) reported that the A/D−/AA model showed the highest response to selection over 15 generations of selection. Both studies had a reasonable initial value of additive-by-additive epistatic variance, which aided variance conversion and increased the initial additive variance; hence, a higher response to selection.

### Limitations of using DL as a genetic model and future directions

While our results highlight the potential of DL genetic simulation models to retain higher genetic variance under directional truncation selection than classical genetic models, several limitations warrant consideration. First, DL approaches come with substantial computational requirements. Training complex neural networks requires large datasets, extensive hyperparameter tuning, and significant computational resources (Chen et al. 2023; Pedrosa et al. 2024), which may limit their immediate application in simulation studies. Second, DL models face challenges of interpretability (Pérez-Enciso and Zingaretti 2019; Chen et al. 2023). Unlike classical genetic models, in which additive, dominance, and epistatic effects can be explicitly partitioned and biologically interpreted, DL models are nonparametric and operate as black boxes, making it difficult to disentangle which genetic components drive the observed retention of variance. This reduced transparency can hinder trust and adoption among breeders. Thirdly, because DL models are nonparametric, the starting variances of genetic components cannot be explicitly specified; therefore, one cannot know the narrow-sense heritability of a simulated trait until it is estimated. Lastly, DL-based models are not plug-and-play; therefore, the models we introduced here should serve only as a guide and should not be used as is when applied to a new dataset, as there is no assurance that they will perform similarly. Moreover, the results in this study are for a single trait (feed efficiency) and a single pig population; although the arguments are general, empirical validation across traits and species is needed.

However, the promising performance of using DL as a genetic simulation model in our study highlights several important avenues for future research. First, validation across other breeding schemes and species is needed to determine the generalizability of these findings. While our study demonstrates advantages under the simulated conditions presented here, different genetic architectures, mating structures, and trait heritabilities may influence findings, warranting empirical testing across diverse breeding contexts. Second, careful attention must be given to implementation considerations. Integrating DL into practical simulation pipelines requires addressing challenges such as limited computational resources and the need for understanding and expertise in managing complex models. Ultimately, bridging simulation-based insights with real-world applications will be crucial to establishing DL as a reliable and scalable tool for modern breeding programs.

## Conclusion

This paper introduces DL as a genetic simulation model and provides guidance on its use. In addition, we show that genetic trait architectures derived from DL models, when sufficiently complex, capture complex interactions that preserve genetic variance, consistent with findings from real-world selection schemes and experiments, whereas simple models show rapid erosion of genetic variance. Lastly, the long-term response to directional truncation selection and inbreeding is sensitive to the assumed genetic model.

## Supplementary Material

jkag187_Supplementary_Data

## Data Availability

This study simulated a pig breeding scheme using a previously published dataset of Topigs Norsvin damline boars as the founder population. The founder dataset is available on Figshare (doi: 10.6084/m9.figshare.30539882). Supplemental material available at G3 online.
