# Supplementary material for: Using deep learning models as a genetic architecture for the simulation of breeding schemes
Source: G3 (Bethesda). 2026 Jul 9;16(9):jkag187. doi: 10.1093/g3journal/jkag187 (PMC13535453; doi:10.1093/g3journal/jkag187)
Supplement: Supplementary file 1 [file jkag187_supplementary_data.zip › Figure_S2_G3-2026-406994.pdf]

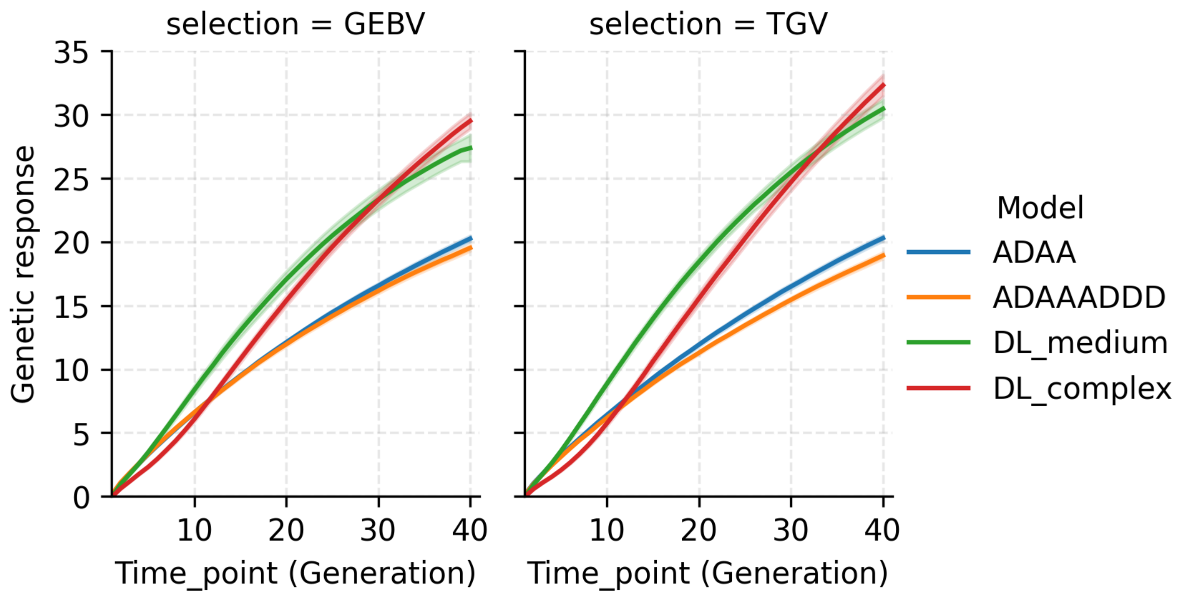

Supplementary Figure 2: Cumulative genetic response for four genetic simulation models under GEBV and TGV selection across 40 generations. The results shown are the averages and standard errors of 10 replicates.
